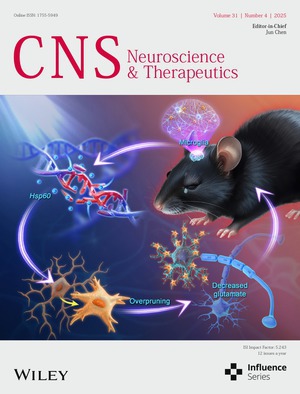# Front Cover

**DOI:** 10.1111/cns.70447

**Published:** 2025-05-21

## Abstract

The cover image is based on the article *Depletion of HSP60 in Microglia Leads to Synaptic Dysfunction and Depression‐Like Behaviors Through Enhanced Synaptic Pruning in Male Mice* by Wenhui Zhu et al., https://doi.org/10.1111/cns.70394.